# Anti-Aβ Antibodies and Cerebral Amyloid Angiopathy Complications

**DOI:** 10.3389/fimmu.2019.01534

**Published:** 2019-07-04

**Authors:** Yannick Chantran, Jean Capron, Sonia Alamowitch, Pierre Aucouturier

**Affiliations:** ^1^Sorbonne Université, Inserm, UMRS 938, Hôpital St-Antoine, AP-HP, Paris, France; ^2^Département d'Immunologie Biologique, Hôpital Saint-Antoine, AP-HP, Paris, France; ^3^Département de Neurologie, Hôpital Saint-Antoine, AP-HP, Paris, France

**Keywords:** cerebral amyloid angiopathy, autoimmunity, anti-Aβ antibodies, immunotherapy, natural antibodies

## Abstract

Cerebral amyloid angiopathy (CAA) corresponds to the deposition of amyloid material in the cerebral vasculature, leading to structural modifications of blood vessel walls. The most frequent form of sporadic CAA involves fibrillar β-amyloid peptide (Aβ) deposits, mainly the 40 amino acid form (Aβ_1−40_), which are commonly found in the elderly with or without Alzheimer's disease. Sporadic CAA usually remains clinically silent. However, in some cases, acute complications either hemorrhagic or inflammatory can occur. Similar complications occurred after active or passive immunization against Aβ in experimental animal models exhibiting CAA, and in subjects with Alzheimer's disease during clinical trials. The triggering of these adverse events by active immunization and monoclonal antibody administration in CAA-bearing individuals suggests that analogous mechanisms could be involved during spontaneous CAA complications, drawing particular attention to the role of anti-Aβ antibodies. However, antibodies that react with several monomeric and aggregated forms of Aβ spontaneously occur in virtually all human individuals, hence being part of the “natural antibody” repertoire. Natural antibodies are usually described as having low-affinity and high cross-reactivity toward microbial components and autoantigens. Although frequently of the IgM class, they also belong to IgG and IgA isotypes. They likely display homeostatic functions and protective roles in aging. Until recently, the peculiar properties of these natural antibodies have hindered proper analysis of the Aβ-reactive antibody repertoire and the study of their implication in CAA complications. Herein, we review and comment the evidences of an auto-immune nature of spontaneous CAA complications, and discuss implications for forthcoming research and clinical practice.

## Introduction

Adaptive immunity in neurodegenerative conditions has recently become of major interest in neurodegenerative conditions, especially in Alzheimer's disease (AD), because of its ability to modulate cerebral inflammation, and also following observations that immunotherapeutic approaches might impair pathological events. However, the nature of immune effectors and their mechanisms remain quite obscure. In this article we will focus mainly on antibodies that bind the β-amyloid peptide (Aβ), which has long been considered a key therapeutic target. Evident difficulties are due to the presence of blood anti-Aβ antibodies in healthy and diseased individuals, implying that most quantitative studies provided little or no information on their pathological implication. Another key point is that effector functions of these antibodies frequently are suggested to result in complications of cerebral amyloid angiopathy (CAA).

## Spontaneously Occurring Anti-Aβ Antibodies in Health and Disease

### Natural Antibodies: Origin, Distinctive Properties and Functions

Natural antibodies were defined as occurring in all normal individuals in the absence of overt antigenic stimulation (1). In spite of many controversies, it is now clear that natural antibodies represent a distinct entity that arises from separate B-cell lineages. Most natural antibodies are produced by a distinct B-cell lineage termed B1 cells, but also in part by marginal zone B cells that belong to the conventional B2 lineage. B1 cells originate very early from precursors of the yolk sac and then from hematopoietic stem cells of the fetal liver, but they also differentiate from bone marrow precursors during the adult life (2).

Classical adaptive B-cell responses to T-dependent antigens feature germinal center reactions that include affinity maturation and isotype switching, resulting in high affinity specific antibodies of IgG, IgA and/or IgE classes. These mechanisms allow improvement of effector efficiency as a function of antigen exposure. At variance with classical antibodies, natural antibodies likely arise from processes that involve no or little B-T cell cooperation and affinity maturation, as suggested by frequent absence or paucity of somatic hypermutations and nucleotide N-additions in the antibody variable regions. Thus, they display quite specific phenotypic properties as compared with conventional antibodies raised by immunization, including low binding affinities and multi-reactivity toward homologous antigens. Interestingly however, they may belong to IgG or IgA, as well as IgM classes (1, 3, 4).

In relation with these peculiar properties, it is thought that natural antibodies display specific functions. They likely protect from infection at early stages of development. In addition to supposed protective roles in the newborn, the ongoing production of natural antibodies has been proposed to interfere with pathological events of adults, such as tumors, atherosclerosis, and neurodegenerative disorders in elderly (5).

Natural IgM antibodies have long been studied in both mice and humans. They display a variety of functions, including pathogen neutralization and killing, and clearance of apoptotic cells and debris that participate in preventing inflammation in several pathological contexts. They also participate in maturation of adaptive responses, especially through antigen recruitment in secondary lymphoid tissues. Other well-known examples of IgM natural antibodies are isohemagglutinins.

Natural IgG and IgA antibodies are also abundant in the blood and mucosal secretions, in spite of the supposed absence of T cell help. Like IgM natural antibodies, they seem to play protective roles, especially in partnership with lectins such as Mannose-Binding Lectin and ficolins, against microbial pathogens and by controlling inflammation (3).

The repertoire of natural antibodies is clearly skewed, as they mostly recognize conserved self-structures (6, 7). Thus, they seem to undergo a selection process (8), suggesting physiological roles (9) including clearance of noxious molecules and cell debris, regulation of immune responses, and shaping of a primary repertoire that allows appropriate induced humoral responses. Numerous observations suggest that natural antibodies are protective against certain pathological conditions of aging, such as atherosclerosis, cancer and neurodegenerative disorders, and that the age-associated decreased expression of these antibodies could be responsible for proneness to these diseases (5). Indeed, circulating anti-alpha-synuclein antibody levels were found to be low in patients with Parkinson's disease (10). However, in AD, anti-Tau natural antibodies expression appears higher than in aged subjects (11). As discussed in the next section, the case of anti-Aβ antibodies and their expression in AD as compared to aged subjects remains controversial.

### Natural Anti-Aβ Antibodies in Healthy Subjects, AD Patients and Related Conditions

Natural antibodies that react with Aβ peptide have long been suggested by the finding of specific B-cells (12), and their invariable presence was confirmed by studies comparing AD patients and healthy subjects (see below). As also found for other natural autoantibodies, aging results in progressive decrease of circulating anti-Aβ levels (13). They belong to both IgM and IgG classes, and predominant IgG subclasses appear to be IgG1 and IgG3 in both healthy and diseased subjects (14). They display a large panel of specificities for both linear and conformational epitopes of the Aβ molecules, oligomers, and fibrillar aggregates, and most interestingly, *in vitro* studies suggested that some natural anti-Aβ antibodies could protect neurons from toxic Aβ oligomers (15).

Analyses performed on serum or plasma from AD patients yielded remarkably incoherent results, as some of them revealed lower anti-Aβ antibody levels as compared with healthy controls (16–19), while others found higher levels in Alzheimer's disease patients (20–22) or no significant difference (13, 14, 23). Analyses performed on cerebrospinal fluid (CSF) led to similar discrepancies, displaying either lower (13, 16) or higher (20, 22) levels of anti-Aβ antibody in AD patients as compared with healthy controls. A common observation of those studies was the striking heterogeneity of antibody titers in healthy as well as in diseased individuals.

A first explanation for inconsistent results may be the diversity of methods used for antibody analysis: coated reactive antigens were Aβ_1−40_ (16, 18, 19) or Aβ_1−42_ (13, 17, 21, 23) in either oligomeric (13, 14, 18), fibrillar (20) or undefined isoforms (16, 17, 19, 21), at extremely variable concentrations; serum or plasma samples were tested either directly (17–20) or after dissociation of immune complexes (13, 14, 21); revealed antibodies were either IgG only (19) or all immunoglobulin isotypes (23); finally, a variety of different quantitation formulas were used. The only methodological agreement between recent studies is the requirement of an initial immune complex dissociation step for a reliable analysis of anti-Aβ antibodies (13, 14, 21), using a mild acidic buffer in order to avoid artifacts (24). Indeed, a large part of circulating anti-Aβ antibodies appears to be associated with a variety of undefined ligands, likely due to polyreactivity and the presence of anti-idiotypic antibodies (15). A possible additional bias is that IgM antibodies have been demonstrated to potentially block natural IgG reactivity (25).

In addition to methodological matters, the other explanations of differences in anti-Aβ titers found between healthy and AD people relied on quite eclectic hypotheses: favored pathology due to decreasing antibody levels in aging (16), sequestering of antibodies by amyloid deposits (16), partial tolerization of B-cell responses (19) or decreased T-cell help (17), onset of an anti-Aβ autoimmune reaction (21, 22), or failure of immune complex clearance (22).

### Natural Antibodies in Therapeutic Trials

That age-related decline of natural autoantibodies favors the onset of pathological conditions related to antigenic targets is supported by strong arguments (3). The hypothesis that natural anti-Aβ antibodies bear a strong protective potential (13, 26) has been the rationale for therapeutic trials in AD using human intravenous immunoglobulins (IVIg). Indeed, IVIg include natural antibodies to Aβ (27, 28) as well as to protein Tau (29). IVIg infusions appeared to increase the levels of circulating anti-Aβ (30), but while preliminary results on mild and moderate stages of AD had proven encouraging, more powerful trials failed to show a significant effect on cognitive decline (31–33). The reason of the lack of clinical efficacy of the natural anti-Aβ antibodies contained in IVIg is a matter of debate. It has been shown that IgG levels were elevated in the CSF of treated subjects. Thus, anti-Aβ antibodies penetrate into the CNS after infusion. Like other Aβ-modifying therapies, IVIg might be more effective in early stages of the disease. In addition, in these trials, IVIg were administered at low dose, similar to antibody replacement therapies. Immunomodulatory effects of IVIg are generally obtained in other neurological disease up to 2 g/kg (33).

## Adaptive Immunity Against Aβ: the AN1792 Trial

Histological response and cognitive improvement after Aβ vaccination in transgenic mouse models of AD have been received enthusiastically in the late 1990's (34, 35). Elan/Wyeth-Ayerst Pharmaceuticals set up the AN1792 trial in human (NCT00021723), the phase I study of safety and immunogenicity began in 1999, Dec., vaccinating mild-to-moderate AD subjects with pre-aggregated Aβ_1−42_ with adjuvant QS-21, and later on with QS-21 plus polysorbate-80 0.4%. After no major safety issue, phase IIa (Study 201) began in 2001, Oct., and was halted in 2002, Jan., when some patients presented with meningoencephalitis (36). Other adverse events other than meningoencephalitis occurred in the AN1792 study: two cases of encephalopathy including one without meningoencephalitis, two cases of convulsion, one thrombosis of the retina, one hemiplegia, and, interestingly, one case of intracerebral hemorrhage (ICH) related to CAA. However, confronted with the striking manifestations of brain inflammation, these post-immunization hemorrhagic or ischemic events have been overlooked (37).

Overall, 18 out of 300 (6%) subjects treated with AN1792 in phase II developed meningoencephalitis, along with 1 subject from the extended phase I study (37–40). Clinically, these patients presented with subacute aseptic meningoencephalitis with confusion, lethargy, and headaches. Other symptoms were nausea, seizures, drowsiness, ataxia, aphasia, hemiparesia, with inconstant fever. As in aseptic meningoencephalitis, CSF analysis showed mild to moderate hyperlymphocytosis (3-130 cells/mm^3^), often with mild hyperproteinorachia (39). Since none of the placebo-treated group developed such serious adverse events, it was obvious that these cases of meningoencephalitis related to the active immunization against Aβ.

Only 2 patients with post-Aβ immunization meningoencephalitis were investigated at the histological level. The first patient (40) presented with fever, drowsiness, and unstable gait. At neuroimaging, MRI found white matter hyperintensities (WMH) and a more focal lesion, possibly associated with edema and inflammatory process. Post-mortem tissue sampling showed cortical areas without senile plaques, but still with CAA, and lymphocytic infiltration of leptomeningeal Aβ-laden blood vessels, or in perivascular space around cortical vessels, with sparse cellular infiltration of the parenchyma. Cellular infiltrates were mainly composed of CD4+ CD45RO+ T-cells, with few CD8+ T-cells, and without CD79a+ CD20+ B-cells. The second patient (41) presented similar plaque-devoid cortical areas and CD8+ T-cell-enriched perivascular lymphocytic infiltrates. In addition, the authors describe multiple macroscopic cortical hemorrhages that were not detected radiologically despite 3 MRI studies, and microscopic vasculopathy, namely lipohyalinosis. Interestingly, a third case (42) reported pathological findings in an AN1792-treated patient without clinical meningoencephalitis, which also presented perivascular cellular infiltrates in the leptomeningeal spaces, though to a lower extent as compared with the meningoencephalitis cases, and composed mainly of CD20+ B-cells and few CD3+ T-cells.

The immune mechanism, underlying these cases of meningoencephalitis has been debated, though it seemed obvious that adaptive immunity against Aβ was at stake.

In the AN1792 trial, treated-patients were considered either antibody responder or non-responder with respect to the circulating anti-Aβ antibody titer supposed to confer clinical benefit (38). While 20% treated patients were considered antibody responders, most patients (at least 90%) seemed to have produced detectable levels of circulating IgM and/or IgG anti-Aβ after immunization (37). Among treated-patients with meningoencephalitis, 13 out of 18 belonged to the responder group, and 5 out of 18 to the non-responder group. Among these 5 non-responders, all of them had a measurable anti-Aβ IgM response, and 4 a measurable anti-Aβ IgG response in the blood (38).

If almost all treated patients developed an anti-Aβ antibody response, one could question whether the antibody response was qualitatively different in patients with meningoencephalitis. However, it was shown by epitope-mapping that all immunized patients recognized a linear B-cell epitope in the N-terminus of the Aβ peptide (Aβ_1−8_), independently of the occurrence of meningoencephalitis (43), and that their serum antibodies bound to parenchymal as well as vascular Aβ aggregates, and not to cellular APP (44).

Out of 57 studied cases, 9 (15%) had measurable CSF IgG or IgM anti-Aβ antibody levels and 4 presented with meningoencephalitis (3 responders and 1 non-responder) (38). However, CSF antibodies from both patients with or without meningoencephalitis bound to senile plaques and vascular Aβ aggregates (44).

T-cell responses during the AN1792 trial have been poorly studied. One paper reports on the higher occurrence of IFNγ producing cells after Aβ_1−42_ stimulation in phase IIa treated-patients as compared to phase I treated-patients, which was not significantly different from the placebo-treated group (45).

Confronted to the absence of noticeable differences in the anti-Aβ antibody responses in AN1792-treated patients with and without meningoencephalitis, and considering the presence of lymphocytic infiltrates during such cases, it was assumed that the adaptive anti-Aβ cellular T-cell response was detrimental when oriented toward cellular immunity through Th1 CD4+ and cytotoxic CD8+ T-cells, while anti-Aβ B-cell response would remain innocuous and potentially beneficial for AD pathology. Thus, subsequent anti-Aβ immunotherapy trials were based on anti-Aβ antibodies either by selective “humoral” vaccines or, most frequently, by passive immunization using repeated humanized monoclonal antibody infusions, the complications of which have been linked to CAA.

## Cerebral Amyloid Angiopathy and Related Clinical Manifestations

### Definition, Epidemiology, and Neuropathological Features of Cerebral Amyloid Angiopathy

Deposited amyloid material in the blood vessel walls of the cerebral vasculature is made up of insoluble fibrils resulting from the polymerization of peptidic subunits with a “crossed β-sheet”-rich conformation. Mutations of several proteins have been described in familial forms of CAA, such as Aβ precursor protein (APP) and other proteins (46). In addition to mutations, duplications of the wild-type *APP* gene are also associated with CAA, as found in Down syndrome and in autosomal dominant early onset Alzheimer's disease (47). Sporadic CAA, which is an Aβ-related disease without any mutation or duplication of the *APP* gene, represents the most common form of CAA and will be the main focus of this review.

The main risk factors of sporadic CAA are advancing age and the co-occurrence of AD (48, 49). Both frequency and severity of CAA increase with age, affecting 35, 50, and 75% of individuals in the 7th, 8th and 9th decades of life, respectively, regardless of CAA severity (49). Population-based studies show that in age-matched groups, demented individuals have higher risk to present CAA than non-demented individuals, regardless of CAA severity or considering solely severe CAA (50). Other genetic risk factors have been described, some of them overlapping with sporadic AD risk factors, making it difficult to distinguish which ones are independently related to sporadic CAA. The main genetic risk factor is ApoE polymorphism: the ε4 allele is associated with the occurrence of AD and CAA, while the ε2 allele appears protective against AD but associated with the risk of CAA-related intra-cerebral hemorrhage (ICH) (51). To explain these controversial findings, Greenberg et al. hypothesized that the APOE2 allele might be related to CAA vasculopathy but not vascular deposits, which they evidenced by showing that the APOE2 allele is more frequent in patients with grade 3-4 CAA (cracking of the vessel wall and paravascular leaking of blood) but that it is not over-represented in grade 2 CAA (complete amyloid replacement of vessels) (52). Hence, APOE4 might promote a higher risk of CAA-related hemorrhage by enhancing amyloid deposition, while APOE2 might cause amyloid-laden vessels to undergo vasculopathic changes that lead to their rupture.

It is indeed important to stress that, from a pathological point of view, CAA does not stand for the mere presence of fibrillary Aβ deposition in blood vessel walls. More severe vascular deposits are associated with vasculopathies, such as fibrinoid necrosis, thrombosis, microhemorrhages, or “double-barreling,” which is the concentric splitting of the vessel walls (53). Associated with smooth muscles cell degeneration and amyloid replacement of the whole vessel wall structure, these pathological features provide the histologic ground for clinical and paraclinical features of the manifestations of CAA. Cerebrovascular amyloid deposition in sporadic CAA involves the leptomeningeal and cortical medium- and small-sized arteries/arterioles, with or without involvement of the capillaries, and usually sparing the veins. The topography of CAA is not homogenous through the brain. The most affected areas being the occipital, parietal, frontal, then temporal lobes. In most advanced cases, other structures than the neocortex can be affected. It is now thought that some CAA features, such as capillary involvement, dyshoric angiopathy hence small vessel disease occurring by capillary occlusion, are associated with AD pathology, while leptomeningeal and arteriolar involvement is mainly independent from parenchymal amyloidosis of AD pathology (49). This could lead to a reappraisal of ApoE polymorphism in CAA: the ε4 allele would be associated with AD capillary CAA, while the ε2 allele would be associated with arterial CAA and its specific manifestations, such as lobar ICH (51).

### CAA Manifestations

Cerebrovascular impairment in sporadic CAA may result in 3 subsets of manifestations: ischemia, bleeding, and inflammation (54).

Ischemic manifestations of CAA are cortical infarcts and WMH appearing on the MRI. They predominate in posterior regions of the brain where CAA accumulates, and relate to hypoperfusion or vessel occlusion (55–57).

Hemorrhagic manifestations of CAA are symptomatic ICH that differentiates from hypertensive ICH by strictly lobar localization, corresponding to the leptomeningeal and cortical topography of CAA (58). Besides symptomatic ICH, cortical microbleeds (59) and superficial cortical siderosis are other hemorrhagic features of CAA that can be displayed on MRI (60). Both ischemic and hemorrhagic features of CAA contribute to chronic vascular dementia.

Inflammatory manifestations of CAA involve a protean clinical presentation of corticosensitive encephalopathy, e.g., with subacute cognitive decline, seizures, headaches, and asymmetric T2 WMH. Histopathologicaly, different inflammatory subtypes have been described associated with CAA: (i) Aβ-related angiitis, which corresponds to destructive transmural infiltrates with granulomatous features; (ii) CAA-related inflammation (CAA-ri), which corresponds to non-destructive perivascular lymphocytic infiltrates. However, besides histopathological phenotypes, clinical features, imaging and outcome largely overlap, making these entities likely part of the same spectrum (61, 62). These pathologies represent up to 30% of primary CNS vasculitis, making it part of single-organ vasculitis in the Chappell-Hill revised nomenclature (63, 64), and introducing the hypothesis of an auto-immune mechanism of these manifestations.

With the noticeable exception of extremely rare CAA-ri cases, the potential auto-immune mechanisms at stake in CAA manifestations have been neglected. Clinical and imaging features of CAA-related hemorrhages or ischemia have been conceived as the consequence of mechanical constrains, blood wall weakening, mural degeneration and or vessel occlusion related to vascular Aβ deposition.

However, it has been convincingly shown that CAA was increased in patients immunized with AN1792. In a post-mortem study of 9 treated-patients, Boche et al. found a worsening of CAA in AN1792 patients, with cerebrovascular deposits containing not only Aβ_1−40_, which is classically found in CAA, but also higher cerebrovascular levels of Aβ_1−42_, the main component of senile plaques. These patients also had a higher rate of vasculopathies and microhemorrhages (65). In our opinion, and as suggested by some authors (66, 67), the possibility cannot be discarded that detrimental adaptive immunity raised against Aβ during the AN1792, whether humoral or cellular, was as least partly related to cerebrovascular deposits, given: (i) the predominant leptomeningeal and perivascular location of cellular infiltrates in meningoencephalitis cases during the AN1792 trial, (ii) the occurrence of some post-immunization hemorrhagic or ischemic severe adverse events, including one case of CAA-related ICH, and (iii) the multiple cortical hemorrhages and associated lipohyalinosis described by Ferrer et al. in one meningoencephalitis case (41).

Whether meningoencephalitis and other adverse events were related to adaptive immune reaction against parenchymal or against vascular Aβ deposits, it was assumed that T-cell anti-Aβ response was detrimental, while humoral adaptive anti-Aβ response was thought to be inocuous and potentially beneficial. However, adverse events also occurred in the course of passive immunotherapies with anti-Aβ antibodies, which shed a new light on the pathophysiology of induced and spontaneously-occurring CAA manifestations.

## Monoclonal Anti-Aβ Antibodies and Their Relationship to CAA Manifestations

### The Illustrative Case of Bapineuzumab

Bapineuzumab is a monoclonal humanized IgG1 antibody corresponding to the murine clone 3D6, targeting the N-terminus of the Aβ peptide (Aβ_1−5_). However, unlike circulating antibodies arising in AN1792 patients, Bapineuzumab does not recognize a linear epitope but a conformational one (68). While patients treated by Bapineuzumab showed a decrease of the cerebral amyloid load by 11C-PiB TEP-scan (69, 70), they did not show clinical improvement (71).

During the phase I trial, 3 out of 10 patients treated with a single infusion of the highest dose of Bapineuzumab (5 mg/kg) developed transient signal abnormalities on T2^*^/fluid attenuation inversion recovery (FLAIR) sequences, interpreted as a sign of vasogenic cerebral edema (72). Additional cases were reported during phase II (73) and phase III (74). First referred as vasogenic edema, these amyloid-related imaging abnormalities (ARIA) were included in a spectrum of so-called ARIA-E (E standing for effusion), that encompasses increased MR signal intensity on FLAIR or other T2-weighted sequences in the parenchyma gray or white matter and/or leptomeninges, sometimes in the cerebellum or brainstem. Interestingly, this radiological presentation appears similar to MRI presentation of spontaneous inflammatory manifestations of CAA (66, 74). ARIA-E was found in 17% (36/210) Bapineuzumab-treated subjects, appearing after the first or second infusion, in a dose-dependent manner, with an increased risk in ApoE ε4 carriers (7% among non-carriers, 18% among heterozygous, 36% among homozygous) (71). ARIA-E was associated with clinical manifestations during Bapineuzumab trials in about 20% subjects. If acute clinical worsening prompted off-protocol MRI in some cases, most patients were detected by per-protocol MRI. However, some were retrospectively found to present clinical signs, similar to inflammatory manifestations of CAA. Another type of ARIA, namely ARIA-H (H standing for hemorrhages) appeared during the Bapineuzumab studies in 12% patients, and featured either microbleeds or cortical superficial siderosis similar to spontaneous hemorrhagic manifestations of CAA. ARIA-E and ARIA-H are not independent phenomena. First, the presence of baseline microbleeds increased the risk of ARIA-E. Second, *de novo* ARIA-H were 10 times more prevalent in patients with ARIA-E (67). They appeared during the same timeline, and often in the same cortical areas (66).

Although no neuropathological description of ARIA-E have been reported, the pathophysiological relationship between ARIA-E and spontaneous CAA-related inflammation on the one hand, and ARIA-H, and spontaneous CAA-related hemorrhages on the other hand, was suggested. Hence, it became obvious that antibodies targeting Aβ are sufficient to trigger CAA-like manifestations, and that T-cell anti-Aβ responses arising from active immunization are not required. Thus, some authors suggested that despite differences in severity, meningoencephalitis and ARIA-E could be part of a same pathophysiological spectrum linked to Aβ vascular deposition (66, 67).

### ARIA in Other Anti-Aβ Passive Immunotherapies

Aducanumab is a human IgG1 selected from a B-cell library obtained from healthy aged donors who were cognitively normal. It was selected for its reactivity against soluble Aβ oligomers and insoluble Aβ fibrils. Similarly to Bapineuzumab, 22% (27/125) Aducanumab-treated subjects developed ARIA-E, 33% being symptomatic, usually mild. Once again, this phenomenon appeared to be dose-dependent and ApoE ε4-related. Among ARIA-E subjects, 15 (56%) also had ARIA-H. In particular, while none of the placebo-treated subjects had cortical superficial siderosis, 9 Aducanumab-treated patients presented with such manifestations (75).

Gantenerumab is a human IgG1 selected by phage display from a human antibody library and optimized *in vitro* to enhance its affinity to fibrillar Aβ, and binds to the N-terminal Aβ_1−10_ but also the middle portion Aβ_19−26_ (76). Then again, 10% (53/531) treated patients developed ARIA-E, and 20% (104/531) developed ARIA-H in a dose-dependent, ApoE ε4-related manner. The clinical course was similar to those of Bapineuzumab and Aducanumab in terms of incidence, symptoms, kinetics and response to withdrawal (77).

Solanezumab is a humanized IgG1 that binds to the mid-domain of soluble Aβ. ARIA-E occurred in 18 Solanezumab-treated subjects, however it was always asymptomatic, with a low incidence (1.1%), and without statistical difference from placebo-treated subjects (0.5%). Under Solanezumab treatment, ARIA-H was more frequent than ARIA-E (9.1%), but once again without statistically significant difference from placebo-treated subjects (7.3%). Even though Solanezumab did not seem to induce a higher frequency of ARIA, the link between ARIA-E and ARIA-H was still obvious: 71% of patients with ARIA-E displayed increases in ARIA-H at the time of ARIA-E, and 48% had co-localized ARIA-H and ARIA-E (78).

Crenezumab is a humanized IgG4 that was designed in order to avoid FcγRc-binding, hence anti-Aβ antibody-mediated cellular reaction in order to lower the risk of ARIA. Crenezumab has a preferential affinity for soluble oligomers (79). Crenezumab-treated patients in the ABBY study did not show a higher incidence of ARIA-E, microbleeds and cortical superficial siderosis. Of note, one asymptomatic macrohemorrhage was reported in a treated-patient (80). In the independent BLAZE study, however, 14.5% treated-patients presented new ARIA-H, as compared to 3.4% placebo-treated patients (81).

Ponezumab is a humanized IgG2 targeting the C-terminus of Aβ_30−40_ that binds to soluble Aβ. The incidence of microhemorrhages was 16.4% in the Ponezumab group and 21.4% in the placebo group. However, various MRI features corresponding to what can be observed during CAA were reported solely in the Ponezumab-treated group, either inflammatory-like (1 case with ARIA-E associated with superficial siderosis, 1 case with cerebral/meningeal enhancement), hemorrhagic-like (1 case of subdural hematoma), or ischemic-like (4 cases of cortical infarcts, 2 subcortical gray-matter infarcts) (82). In 24 CAA patients treated with Ponezumab, 2 hemorrhagic adverse events (1 case of cerebral hemorrhage, 1 case of subdural hematoma), and 1 inflammatory-like event (1 case of asymptomatic ARIA-E) occurred but were not considered treatment-related by the investigators (83).

Other antibodies have been used in Aβ-related diseases. AAB-003 was developed by introducing a 3-aminoacid mutation in the Fc region of Bapineuzumab, resulting in reduced FcγRc binding and reduced binding to C1q. ARIA-H and ARIA-E were however reported in the treated group (84). BAN2401 is a humanized IgG1 monoclonal antibody that selectively binds to Aβ soluble protofibrils and that did not appear to be related with ARIA during phase I (85).

## Conclusion: Implications for the Management of CAA Complications

Overall, treatments of Aβ-related diseases with monoclonal anti-Aβ antibodies can trigger CAA-like manifestations, either inflammatory or hemorrhagic. In most cases, these radiological signs are not associated with clinical manifestations. Since they were followed by treatment discontinuation in most trials, the question of whether the asymptomatic radiological signs would have remained infra-clinical or represent a pre-clinical state remains unanswered. The mechanism of such CAA-like manifestations is also unknown. Anti-Aβ antibodies could simply worsen CAA (65) by displacing parenchymal Aβ to the cerebral vasculature, hence enhancing the risk of CAA manifestations. A more direct pathogenic role for anti-Aβ antibodies would involve the mediation of inflammation and/or the triggering of hemorrhagic events after antibody binding to cerebrovascular deposits, by impairment of the structural integrity of the blood vessel, complement-mediated or FcγRc-mediated inflammation. As a general rule, three concomitant factors seem to modulate the risk of ARIA. The dose-dependent effect of such anti-Aβ monoclonal antibodies is the first and better established one. The second one is anti-Aβ antibody isotype: IgG1 anti-Aβ monoclonal antibodies such as Bapineuzumab, Aducanumab, and Gantenerumab, triggered ARIA-E and ARIA-H, while IgG2 (Ponezumab) and IgG4 (Crenezumab) appeared less prone to trigger CAA-like manifestations. The third one is the selectivity for soluble or deposited forms of Aβ: Ponezumab and Crenezumab were more selective for targeting soluble forms of Aβ, as were Solanezumab and BAN2401, hence maybe less prone than other antibodies to bind to cerebrovascular deposits in body fluids.

In any case, the pathogenicity of anti-Aβ antibodies likely depends on their characteristics: isotype (Ig class and subclass), specificity toward soluble or aggregated forms of Aβ_1−42_ or Aβ_1−40_, differential affinity for such forms, and concentration in body fluids.

By analogy with passive immunotherapy-induced ARIA, these considerations lead to the hypothesis that in sporadic CAA, the occurrence of some species of anti-Aβ antibodies can trigger spontaneous inflammatory or hemorrhagic CAA manifestations. However, serum anti-Aβ antibodies have been scarcely studied in CAA (86). Most investigations focused on (i) CSF antibodies (ii) during CAA-related inflammation. Piazza et al. showed elevated CSF levels of anti-Aβ IgG during the clinical and radiological active phase of spontaneous CAA-ri (87). The same group argued in favor of an intrathecal synthesis of CSF anti-Aβ antibodies, based on a single case (88). However, experimental evidence in transgenic mouse model that exhibit AD-like pathology with a strong CAA component suggested that blood anti-Aβ antibodies are by themselves prone to aggravate CAA and related manifestations, including hemorrhages (89, 90).

Anti-Aβ antibodies circulating in the serum are part of the natural antibody repertoire. Despite methodological difficulties hindering their study, adverse events that occurred during anti-Aβ immunotherapies in AD suggest that natural anti-Aβ antibodies are likely to play a role in the triggering of CAA-related inflammation, but also in other CAA manifestations such as hemorrhagic features. Whether such pathogenic anti-Aβ species would be part of an individual pre-immune natural repertoire, or would be the result of adaptive processes driven by cerebrovascular deposits during the course of CAA is unknown. The pathogenic features of these anti-Aβ antibodies that would correlate with CAA clinical phenotypes remain to be determined by a fine-scale analysis of the circulating anti-Aβ antibody repertoire, focusing on serum concentration, isotypes and IgG subclasses, specificity, and affinity toward soluble or aggregated forms of Aβ. Such analysis would deliver pathophysiological insights about CAA manifestations, but would hopefully provide new biomarkers for the prediction of spontaneous and immunotherapy-induced complications in sporadic CAA and AD.

To date, cognition and education remain the best predictors of dementia in the elderly, while ApoE polymorphisms consistently predict Aβ-related pathology (91). The vascular component in AD and other dementia has raised more and more interest over the last years. In this view, the study of spontaneously produced pathogenic anti-Aβ antibodies seems promising. Its inclusion in prediction models of dementia might be the keystone that would link cerebrovascular Aβ deposition and macro- or microvascular impairment.

## Author Contributions

YC and PA wrote the manuscript. All authors revised the manuscript.

### Conflict of Interest Statement

The authors declare that the research was conducted in the absence of any commercial or financial relationships that could be construed as a potential conflict of interest.
